# Intratumoral microbiota stratifies glioma malignancy and patient survival through metabolic associations

**DOI:** 10.3389/fcimb.2026.1940097

**Published:** 2026-10-23

**Authors:** Yao Yao, Yuyan Yang, Jiaqin Song, Li Wu, Mingyue Xu, Haiyao Liu, Yih-Cherng Liou, Lei Xia

**Affiliations:** 1 Department of Cancer Center, The Second Affiliated Hospital of Chongqing Medical University, Chongqing Key Laboratory of Immunotherapy, Chongqing, China; 2 Department of Biological Sciences, Faculty of Science, National University of Singapore, Singapore, Singapore; 3 Integrative Sciences and Engineering Programme, National University of Singapore Graduate School, National University of Singapore, Singapore, Singapore

**Keywords:** glioma, intratumoral microbiota, metabolomics, microecological regulators, overall survival, WHO grading

## Abstract

**Background:**

Glioma is the most common primary malignant tumor of the central nervous system, and its clinical outcomes remain highly heterogeneous despite advances in molecular classification and therapeutic strategies. Emerging evidence suggests that the intratumoral microbiome may influence tumor progression and the tumor microenvironment; however, its role in glioma malignancy stratification and prognosis remains poorly understood. This study aimed to characterize the intratumoral microbiota of glioma tissues and investigate its associations with tumor grade, overall survival, and potential metabolic regulatory mechanisms.

**Methods:**

A total of 107 treatment-naïve glioma patients were retrospectively enrolled, and tumor tissue samples were subjected to 16S rRNA gene sequencing to profile intratumoral microbial communities. Microbial sequence processing and taxonomic annotation were performed using QIIME2 and DADA2 pipelines. Microbial diversity and compositional differences were evaluated using alpha diversity analysis, Bray–Curtis distance-based principal coordinate analysis (PCoA), and differential abundance analysis. Kaplan–Meier survival analysis, univariate Cox regression, random forest feature selection, and multivariate prognostic modeling were performed to identify microbiome-associated survival indicators. Bootstrap optimism correction was performed with 1,000 resamples. PICRUSt2 was used to predict microbial functional profiles.

**Results:**

We found that intratumoral microbiota composition was significantly associated with WHO grade and overall survival. Positively associated taxa, including *Firmicutes* and *Clostridia*, were enriched in low-grade gliomas and favorable-prognosis groups, whereas negatively associated taxa such as *Enterobacterales* and *Porphyromonas gingivalis* predominated in high-grade gliomas and poor-survival patients. The model based on WHO grade–associated microbiota achieved optimism-corrected AUCs of 0.71, 0.86, and 0.88 at 1, 2, and 3 years, respectively. Functional prediction suggested differences in microbial pathways related to nucleotide biosynthesis and amino acid metabolism between groups.

**Conclusions:**

Collectively, these findings suggest associations of intratumoral microbial signatures with glioma malignancy and prognosis, alongside differences in predicted microbial functional profiles. These exploratory observations warrant independent validation to clarify their biological and potential clinical relevance.

## 1 Introduction

Glioma is the most common primary tumor of the central nervous system and remains associated with poor clinical outcomes. According to the World Health Organization (WHO) classifications, gliomas are stratified into low-grade (grade I/II) and high-grade (grade III/IV) tumors, with prognosis tightly linked to tumor grade. Patients with WHO grade IV glioma typically exhibit a median survival of less than two years despite aggressive multimodal therapy (Yang et al., 2022).

In recent years, glioma diagnosis and management have transitioned toward a molecularly informed precision medicine framework. The WHO Classification of Tumors of the Central Nervous System, 5th Edition (WHO CNS5), incorporates key molecular features, including isocitrate dehydrogenase (IDH) mutation status, and O^6^-methylguanine-DNA methyltransferase (MGMT) promoter methylation, CDKN2A/B homozygous deletion, telomerase reverse transcriptase (TERT) promoter mutation, epidermal growth factor receptor (EGFR) alterations, and chromosomal +7/-10 copy number changes, to refine tumor classification and prognosis stratification (Weller et al., 2024). However, substantial heterogeneity in clinical outcomes persists even among patients with similar molecular subtypes, implying the potential involvement of additional biological determinants that are not captured by current classification systems.

The tumor microbiome, often referred to as the “second genome,” has emerged as a previously underappreciated component of the tumor microenvironment. Accumulating evidence indicates that microorganisms reside within multiple solid tumors and influence cancer progression by modulating immune responses, metabolic pathways and therapeutic sensitivity (Nejman et al., 2020). Recent studies have further confirmed the presence of microbial DNA and even viable bacteria in brain tumors, with their distribution exhibiting tumor type- and location-specific patterns (Gigi et al., 2025; Morad et al., 2025). This finding not only challenges the long-held notion of the central nervous system as a “sterile fortress” but also introduces new considerations for the current precision medicine framework grounded in molecular signatures. Nevertheless, systematic investigations examining the clinical relevance of intratumoral microbiota in glioma, particularly in relation to WHO grade, clinical prognosis and patient survival, remain limited.

In this study, we employed 16S rRNA high-throughput sequencing on 107 treatment-naïve glioma specimens and integrated microbial profiles with clinical and survival data. By combining machine learning-based modeling with functional pathway prediction, we aimed to characterize intratumoral microbial signatures associated with glioma malignancy and prognosis and to explore their potential biological and translational implications.

## 2 Materials and methods

### 2.1 Patient cohort and sample collection

Formalin-fixed paraffin-embedded (FFPE) glioma specimens were retrospectively obtained from the pathological archives of the Second Affiliated Hospital of Chongqing Medical University following approval by the Institutional Review Board. The study was approved by the Ethics Committee of the Second Affiliated Hospital of Chongqing Medical University (Approval number: No.(2026)573).

Patients were eligible if they had a histopathological diagnosis of primary glioma (WHO grades I–IV), underwent surgical resection without receiving preoperative radiotherapy, chemotherapy, or targeted therapy, and had complete clinical follow-up records. Patients were excluded if they presented with secondary or recurrent gliomas, prior anti-tumor treatment, concurrent malignancies or systemic infectious diseases (including HIV or active hepatitis), insufficient follow-up information, poor-quality tissue samples, failed DNA extraction, or a history of severe autoimmune disease or long-term immunosuppressive therapy.

A total of 107 treatment-naïve glioma samples fulfilled the inclusion criteria and were included in the final analysis. Haematoxylin and eosin (H&E)-stained sections were independently reviewed by experienced neuropathologists to confirm tumor diagnosis and grading (Weller et al., 2024). Clinical data collected included age at diagnosis, sex, date of surgery, histological subtype, WHO grade, survival status, and date of death or last follow-up. Overall survival (OS) was defined as the interval between the initial surgical resection and death or last follow-up. tumor grading was assigned according to the 2021 WHO Classification of tumors of the Central Nervous System (Louis et al., 2021).

### 2.2 DNA extraction from FFPE tumor tissues

tumor tissues obtained during surgery were preserved immediately and subsequently processed by formalin fixation and paraffin embedding. Genomic DNA was extracted using the cetyltrimethylammonium bromide (CTAB) method (Paireder et al., 2013). Briefly, FFPE tissue sections were incubated in CTAB lysis buffer supplemented with lysozyme and heated at 65 °C to promote cellular disruption. DNA purification was performed by sequential extraction with phenol–chloroform–isoamyl alcohol (25:24:1) followed by chloroform–isoamyl alcohol (24:1). Nucleic acids were precipitated using isopropanol, washed with 75% ethanol, air-dried, and resuspended in double-distilled water. Residual RNA was removed by RNase A treatment at 37 °C to obtain high-quality genomic DNA for downstream analysis.

### 2.3 PCR amplification and library preparation

Bacterial profiling was primarily performed using primers targeting the V4 hypervariable region of the 16S rRNA gene (515F: GTGCCAGCMGCCGCGGTAA; 806R: GGACTACHVGGGTWTCTAAT).

PCR amplification was carried out using Phusion High-Fidelity PCR Master Mix (Thermo Fisher Scientific, Waltham, MA, USA). Nuclease-free-water PCR negative controls were included during amplification, but were not sequenced. Each reaction mixture contained 15 μL master mix, 0.2 μM of each primer, and 10 ng of template DNA. The amplification program consisted of initial denaturation at 98 °C for 1 min, followed by 30 cycles of denaturation at 98 °C for 10 s, annealing at 50 °C for 30 s, extension at 72 °C for 30 s, and a final extension at 72 °C for 5 min.

PCR products were purified using magnetic beads supplied by Novogene Co., Ltd. (Beijing, China) and pooled at equimolar concentrations. Sequencing libraries were constructed following standard protocols and quantified using Qubit 2.0 Fluorometer (Thermo Fisher Scientific, Waltham, MA, USA) and quantitative PCR on a QuantStudio 12K Flex Real-Time PCR System (Thermo Fisher Scientific, Waltham, MA, USA). Libraries that passed quality control were sequenced on an Illumina NovaSeq 6000 system (Illumina, San Diego, CA, USA; Bokulich et al., 2013).

### 2.4 Low-biomass contamination control and quality assurance

Given the low microbial biomass associated with intratumoral tissue samples, stringent contamination control procedures were implemented throughout experimental processing. Paraffin-only blank samples and DNA extraction negative controls (no-tissue controls) were included in parallel during DNA extraction, library preparation, and sequencing to monitor potential background contamination. A paraffin-only blank relative-abundance profile is provided in **Supplementary Table 1**.

All laboratory procedures were conducted using sterile, DNA-free consumables under aseptic conditions. Reagents from identical production batches were used whenever possible to minimize batch-related variability. Sequencing reads detected in negative controls were systematically compared with biological samples. Amplicon sequence variants (ASVs) showing dominant abundance in negative controls or exhibiting background-level distribution patterns were excluded prior to downstream analysis. In addition, low-prevalence and extremely low-abundance ASVs were filtered to reduce potential artifacts associated with reagent contamination or index hopping. These combined measures were applied to improve data reliability and preserve biologically meaningful microbial signals.

### 2.5 Bioinformatic processing and data quality control

Raw sequencing data were demultiplexed based on sample-specific barcodes and primer sequences. Paired-end reads were merged using Fast Length Adjustment of SHort reads (FLASH; version 1.2.11; Magoč and Salzberg, 2011) to generate raw sequence tags. Primer sequences were trimmed using Cutadapt to remove residual amplification artifacts. Quality filtering was subsequently performed using fastp (version 0.23.1) to retain high-quality reads based on read length, base quality, and sequence integrity.

Filtered reads were aligned against the SILVA reference database to identify and remove chimeric sequences generated during PCR amplification. Only high-quality, non-chimeric reads were retained for downstream analyses. Negative control datasets were processed in parallel using the same bioinformatic pipeline to facilitate contaminant screening and quality assessment.

### 2.6 ASV denoising and taxonomic annotation

Taxonomic annotation was performed against SILVA 138.1 using QIIME2 (Singh and Wahengbam, 2026) classify-sklearn with a pretrained naive Bayes classifier and a confidence threshold of 0.7. ASV assignments and classifier confidence values are provided in **Supplementary Table 2**, and representative sequences in **Supplementary Material 7**.

## 3 Results

### 3.1 Intratumoral microbiome landscape in glioma tissues

Microbial profiling was performed on 107 glioma specimens to characterize the intratumoral microbiome. The relative abundances of the top 10 taxa across taxonomic levels (phylum to species) are summarized in **Supplementary Figure 1**. At the phylum level (**Figures 1A, B**), *Proteobacteria, Firmicutes, Actinobacteriota, and Bacteroidetes* constituted the dominant microbial populations. In addition to these common taxa, several low-abundance phyla, including *Spirochaetota* and *Crenarchaeota*, were consistently detected, suggesting selective microbial adaptation within the glioma microenvironment. At lower taxonomic levels, enrichment of genera such as *Escherichia–Shigella* and *Streptococcus* was detected, potentially reflecting altered blood–brain barrier permeability or abnormal microbial infiltration into the tumor niche.

**Figure 1:**
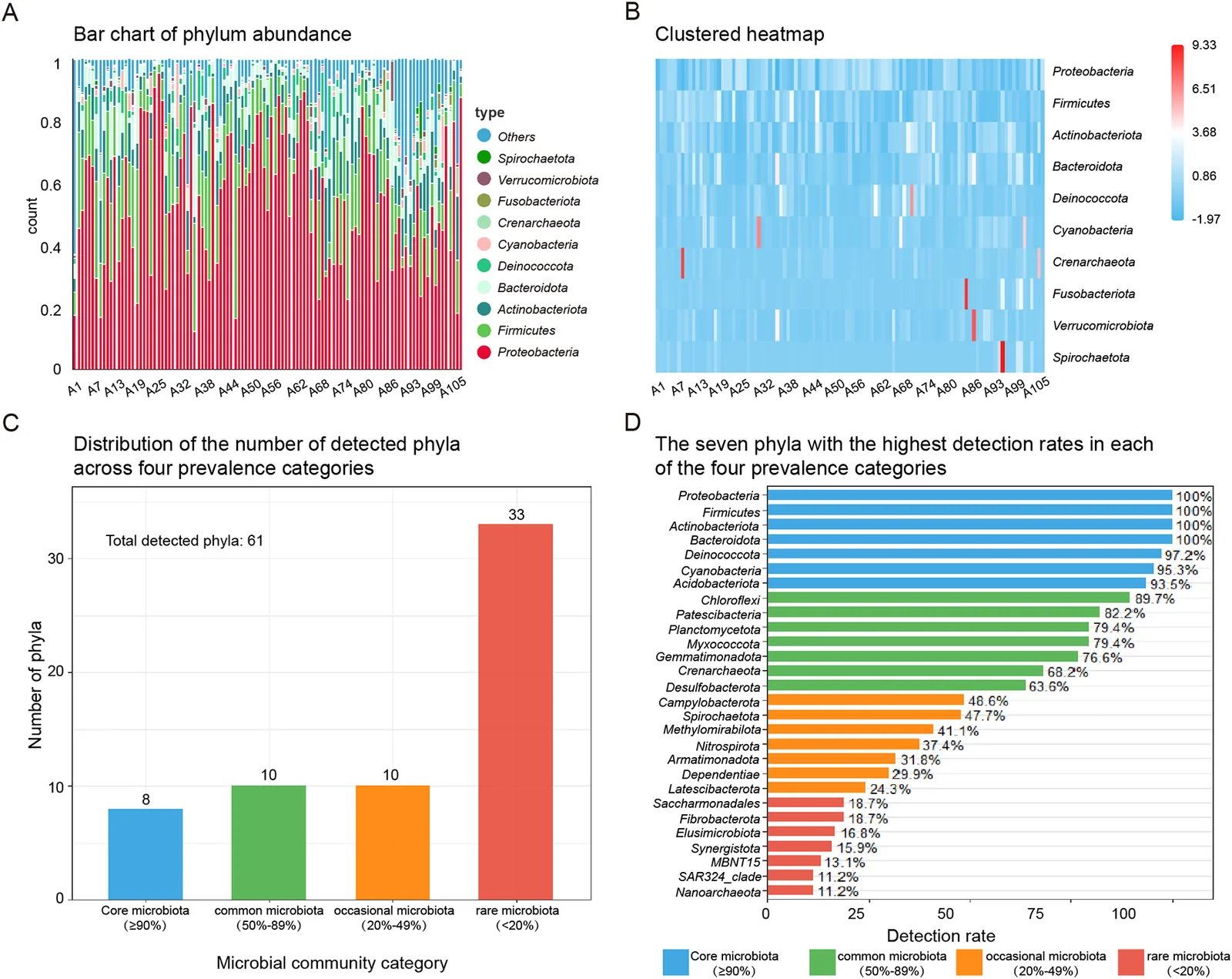
Quantitative profiling of dominant microbial taxa in 107 glioma samples. **(A)** Bar plot of the relative abundance of the top 10 dominant taxa in each sample. **(B)** Heatmap of log-transformed abundance with hierarchical clustering of samples. **(C)** Distribution of the number of detected phyla across four prevalence categories. **(D)** The seven phyla with the highest detection rates in each of the four prevalence categories.

Across the 107 samples, a total of 61 microbial taxa at the phylum level were detected. These taxa were categorized into four groups based on their detection rates (**Figures 1C, D**). Core microbiota, including *Proteobacteria* and *Firmicutes* were detected in more than 90% of samples. Notably, the rare microbiota encompassed the largest number of microbial phyla, comprising 33 of the 61 identified phyla (54.1%), highlighting substantial microbial heterogeneity within the glioma microenvironment.

Together, these findings indicate that glioma tissues harbor distinct intratumoral microbial communities with compositional features that differ from those typically observed in non-tumor environments.

### 3.2 Intratumoral microbiota composition differs between prognostic groups

To evaluate the relationship between intratumoral microbiota and clinical outcome, overall survival (OS) data from all 107 patients were analyzed (**Table 1**). Optimal cut-off analysis identified 664 days as the threshold that maximized survival separation (P < 0.001), stratifying patients into a favorable-prognosis group (n = 37) and a poor-prognosis group (n = 70) (**Figures 2A, B**).

**Table 1:** Baseline characteristics of patients.

Characteristics	Overall (n = 107)	Survivors (n = 39)	Deceased (n = 68)	P value
Age (in years)	53 (14-84)	45.36 ± 17.56	54.01 ± 13.97	0.006
Male, n (%)	63 (58.9%)	17 (43.6%)	46 (67.7%)	0.015
body mass index (BMI)	21.99 (16.65-33.26)	22.99 ± 3.15	22.58 ± 2.59	0.465
WHO Grading				
Grade I, n (%)	2 (1.9%)	2 (5.1%)	0	0.001
Grade II, n (%)	24 (22.4%)	16 (41.0%)	8 (11.8%)	0.001
Grade III, n (%)	26 (24.3%)	8 (20.5%)	18 (26.5%)	0.001
Grade IV, n (%)	55 (51.4%)	13 (33.3%)	42 (61.8%)	0.001
Laboratory test data				
White blood cell count (×10^9^/L)	10.27 (3.82-23.52)	10.01 ± 3.41	11.81 ± 4.83	0.042
Absolute lymphocyte count (×10^9^/L)	0.94 (0.23-2.83)	1.20 ± 0.60	1.00 ± 0.52	0.068
Absolute monocyte count (×10^9^/L)	0.4 (0.02-1.68)	0.33 ± 0.24	0.53 ± 0.34	0.002
Neutrophil count(×10^9^/L)	9.13 (2.13-21.34)	8.44 ± 3.39	10.25 ± 4.71	0.038
C-reactive protein (CRP, mg/L)	5 (0.5-200)	7.85 ± 10.17	11.38 ± 27.9	0.449
Serum C-reactive protein (sCRP, mg/L)	2.02 (0.5-5)	2.53 ± 1.89	2.61 ± 1.90	0.834
Alanine aminotransferase (ALT)	17 (5-119)	25.77 ± 23.05	26.43 ± 22.08	0.884
Aspartate aminotransferase (AST)	19 (9-58)	21.54 ± 11.31	20.87 ± 9.81	0.748
Gamma-glutamyl transferase (GGT)	20 (6-169)	30.23 ± 27.96	30.99 ± 31.55	0.902
Total bilirubin	8.75 (2.5-32.8)	10.03 ± 5.66	9.47 ± 4.55	0.578
Total cholesterol (TC)	4.64 (3.38-7.13)	4.89 ± 0.77	4.76 ± 0.97	0.667
Triglyceride (TG)	1.24 (0.59-4.06)	1.50 ± 0.94	1.50 ± 0.69	0.984
Low-density lipoprotein cholesterol (LDL-C)	2.41 (1.47-3.91)	2.69 ± 0.69	2.54 ± 0.65	0.495
High-density lipoprotein cholesterol (HDL-C)	1.28 (0.69-2.19)	1.36 ± 0.38	1.30 ± 0.36	0.682

Continuous variables with a normal distribution are presented as mean ± standard deviation and were compared using independent-samples t-tests. Categorical variables are presented as frequencies (percentages), and were compared using the chi-square test. A P-value < 0.05 was considered statistically significant. Lipid data were available for 37 patients.

**Figure 2:**
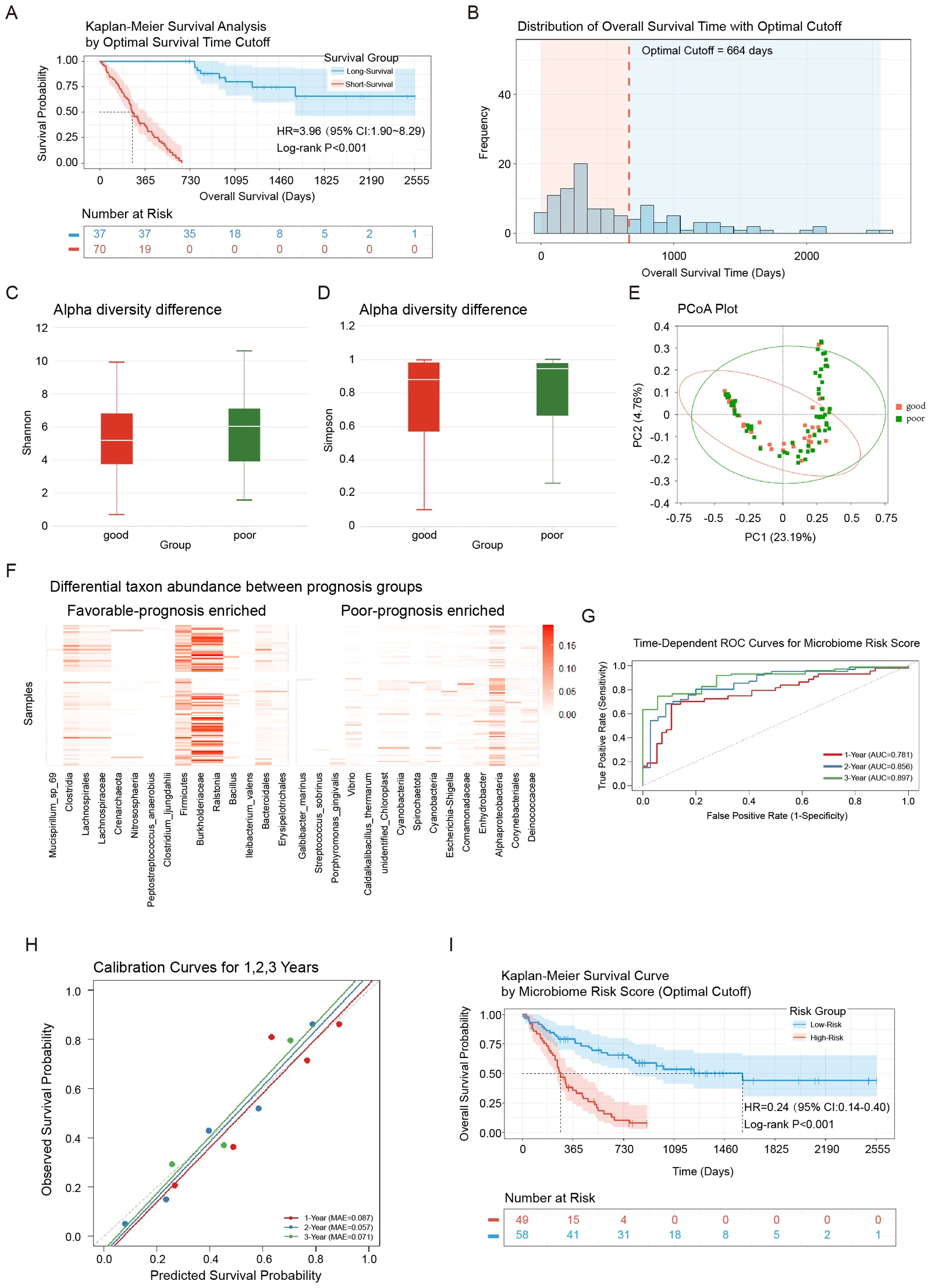
Microbiome-associated survival stratification in glioma. **(A)** Kaplan–Meier survival curves comparing microbiome-defined good and poor prognosis groups using the optimal cut-off determined by maximally selected rank statistics. Hazard ratio (HR)≈3.96, 95% confidence interval (CI):1.90~8.29, and log-rank P <0.001. **(B)** Bar chart of overall survival distribution based on the optimal cut-off value. **(C, D)** Alpha diversity indices (Shannon and Simpson) compared between prognosis groups using the Wilcoxon rank-sum test. **(E)** Principal coordinate analysis (PCoA) based on Bray–Curtis distance showing beta diversity differences between groups. **(F)** Heatmap of differentially abundant taxa across prognosis groups. **(G)** Time-dependent ROC analysis evaluating the predictive performance of the microbiome-based stratification for overall survival. The AUC values were 0.78, 0.86, and 0.90 at 1, 2, and 3 years, respectively. **(H)** Calibration Curves for 1-, 2-, and 3-year survival. **(I)** Kaplan-Meier survival curve based on microbiome-derived risk score (Optimal Cutoff).

Alpha diversity analysis demonstrated significantly higher microbial diversity in the poor-prognosis group compared with the favorable-prognosis group, as reflected by both Shannon and Simpson indices (**Figures 2C, D**).

Principal coordinate analysis (PCoA) based on Bray–Curtis dissimilarity showed overlapping profiles between the favorable- and poor-prognosis groups (**Figure 2E**). PERMANOVA did not detect a statistically significant overall compositional difference (pseudo-F=1.6493, R²=0.0155, P = 0.0505; 9,999 permutations). PERMDISP indicated unequal dispersion between the groups (F = 6.8390, P = 0.0102), further limiting interpretation of the apparent clustering as a difference in group location (**Supplementary Table 5**).

Differential abundance analysis identified distinct microbial signatures associated with survival outcomes. Taxa enriched in the favorable-prognosis group included members of *Firmicutes, Clostridia, Lachnospirales, Lachnospiraceae*, and several short-chain fatty acid–producing bacterial groups (**Figure 2F**). In contrast, the poor-prognosis group was characterized by enrichment of taxa commonly associated with pathogenic or pro-inflammatory profiles, including *Porphyromonas gingivalis, Escherichia–Shigella, Streptococcus sobrinus, Vibrio species*, and *Alphaproteobacteria*.

These results indicate that intratumoral microbiome composition is closely associated with patient survival and reveals distinct microbial patterns between prognostic subgroups.

#### 3.2.1 Microbiota-informed prognostic modeling identifies *Firmicutes* and *Clostridia* as positively associated features

Given the prominent differences observed in *Firmicutes* and *Clostridia* abundance between prognostic groups, we next evaluated their prognostic relevance in the context of clinical variables. Additional clinical data, including anthropometric indices, inflammatory markers, liver function parameters, lipid profiles (n = 37), and molecular information (n = 24), were incorporated for exploratory survival analysis.

Univariate survival analysis showed significant associations between OS and age, sex, body weight, BMI, inflammatory indices, liver function parameters, and lipid profiles (**Table 2**). In contrast, molecular variables, including IDH mutation status, CDKN2A deletion, 1p/19q co-deletion, MGMT promoter methylation, EGFR alterations, and +7/−10 copy number changes, did not reach statistical significance in this subset, likely due to limited sample size (**Table 3**).

**Table 2:** Univariate survival analysis of clinical variables and overall survival (OS) (n = 107).

Variable	Unit	Median (Range) or n (%)	Hazard Ratio (HR) (95% confidence interval, CI)	P - value	Significance
Age	years	53 (14-84)	1.027 (1.010-1.044)	0.001	*
Gender	/	male (n=63) female (n=44)	2.008 (1.192-3.383)	0.008	*
Height	M	1.62 (1.48-1.78)	8.821 (0.168-462.017)	0.467	NS
Weight	kg	59 (40-100)	0.996 (0.969-1.024)	0.002	*
BMI	kg/m2	21.99 (16.65-33.26)	0.956 (0.865-1.056)	0.001	*
White Blood Cell Count	109/L	10.27 (3.82-23.52)	1.052 (0.991-1.116)	0.001	*
Absolute Lymphocyte Count	109/L	0.94 (0.23-2.83)	0.620 (0.388-0.990)	0.001	*
Lymphocyte Percentage	/	8.6 (1.5-38.9)	0.973 (0.940-1.008)	0.001	*
Absolute Monocyte Count	109/L	0.4 (0.02-1.68)	2.753 (1.373-5.517)	0.001	*
Monocyte Percentage	/	4.2 (0.3-13.7)	1.090 (0.999-1.189)	0.001	*
Absolute Neutrophil Count	109/L	9.13 (2.13-21.34)	1.056 (0.993-1.122)	0.001	*
Neutrophil Percentage	/	87.3 (53.1-97.1)	1.009 (0.983-1.035)	0.001	*
CRP	mg/L	5 (0.5-200)	1.020 (1.008-1.032)	0.001	*
sCRP	mg/L	2.02 (0.5-5)	1.041 (0.916-1.183)	0.001	*
ALT	U/L	17 (5-119)	1.004 (0.993-1.014)	0.001	*
AST	U/L	19 (9-58)	1.002 (0.980-1.025)	0.003	*
GGT	U/L	20 (6-169)	1.003 (0.994-1.011)	0.003	*
Total Bilirubin	μmol/L	8.75 (2.5-32.8)	0.993 (0.945-1.043)	0.001	*
Direct Bilirubin	μmol/L	3.2 (1.5-8.9)	1.009 (0.877-1.160)	0.001	*
Indirect Bilirubin	μmol/L	5.35 (0.8-24.3)	0.987 (0.926-1.051)	0.001	*
Total Cholesterol (TC)	mmol/L	4.64 (3.38-7.13)	0.991 (0.596-1.646)	0.001	*
Triglyceride (TG)	mmol/L	1.24 (0.59-4.06)	1.065 (0.647-1.751)	0.001	*
Low-density Lipoprotein Cholesterol (LDL-C)	mmol/L	2.41 (1.47-3.91)	0.863 (0.458-1.626)	0.001	*
High-density Lipoprotein Cholesterol (HDL-C)	mmol/L	1.28 (0.69-2.19)	0.952 (0.292-3.110)	0.001	*

*P < 0.05 indicates statistical significance. NS, not significant (*P* ≥ 0.05). Hazard ratios (HRs) and *P* values were derived from the Kaplan-Meier analysis (Log-rank test) or the Cox proportional hazards model. Lipid data were available for 37 patients.

**Table 3:** Univariate survival analysis of molecular markers and overall survival (OS) (n = 24).

Variables	Mutation/positive rate	P - value	Significance
IDH1 mutation	3(12.5%)	0.104	NS
CDKN2A deletion	7(29.2%)	0.298	NS
1p/19q codeletion	1(4.2%)	0.361	NS
MGMT methylation	6(25%)	0.341	NS
EGFR amplification or mutation	2(8.3%)	0.798	NS
Copy number alterations of +7/−10	8(33.3%)	0.351	NS

NS, not significant (P ≥ 0.05). The relatively small sample size (n = 24) may limit statistical power. Mutation frequencies were calculated based on the number of samples with valid detections (e.g., the IDH mutation rate was 12.5%).

For samples with lipid data, random forest analysis was first applied to reduce dimensionality and identify the most informative predictors. Subsequent LASSO regression identified *Clostridia* as the strongest positively associated microbial feature associated with OS, followed by *Firmicutes*. Lipid-related variables did not remain significant predictors in the final model (**Supplementary Figures 2A–D**).

#### 3.2.2 Integrated microbiome–clinical model predicts glioma survival

To construct a more robust prognostic model, analyses were extended to the full cohort of 107 patients after excluding lipid variables. Random forest screening was first used to select the top 30 survival-associated features, which were subsequently incorporated into LASSO regression for model construction.

The final model identified *Firmicutes* (coefficient = −0.2462) as the strongest positively associated microbial predictor, followed by *Clostridia* (−0.1300), while age, sex, *Galbibacter marinus, Alphaproteobacteria, and Porphyromonas gingivalis* were retained as risk-associated variables (**Supplementary Figures 2E–H**). Time-dependent receiver operating characteristic (ROC) analysis yielded apparent AUCs of 0.78, 0.86, and 0.90 at 1, 2, and 3 years, respectively (**Figure 2G**). Calibration analysis indicated good agreement between predicted and observed survival probabilities (**Figures 2H, I**). After bootstrap optimism correction, the OS-associated model yielded 1-, 2-, and 3-year AUCs of 0.7005, 0.8723, and 0.9591, respectively (**Supplementary Table 3**).

Together, these results demonstrate that integration of intratumoral microbiome features with clinical parameters enables effective stratification of survival risk in glioma patients.

#### 3.2.3 Functional prediction reveals metabolic pathways associated with poor prognosis

To explore the potential functional implications of prognostic microbial signatures, PICRUSt2-based pathway inference was performed. Comparative analysis between favorable- and poor-prognosis groups revealed significant enrichment of pathways related to energy metabolism, nucleotide biosynthesis, and amino acid metabolism in patients with poor prognosis (**Figure 3A**).

**Figure 3:**
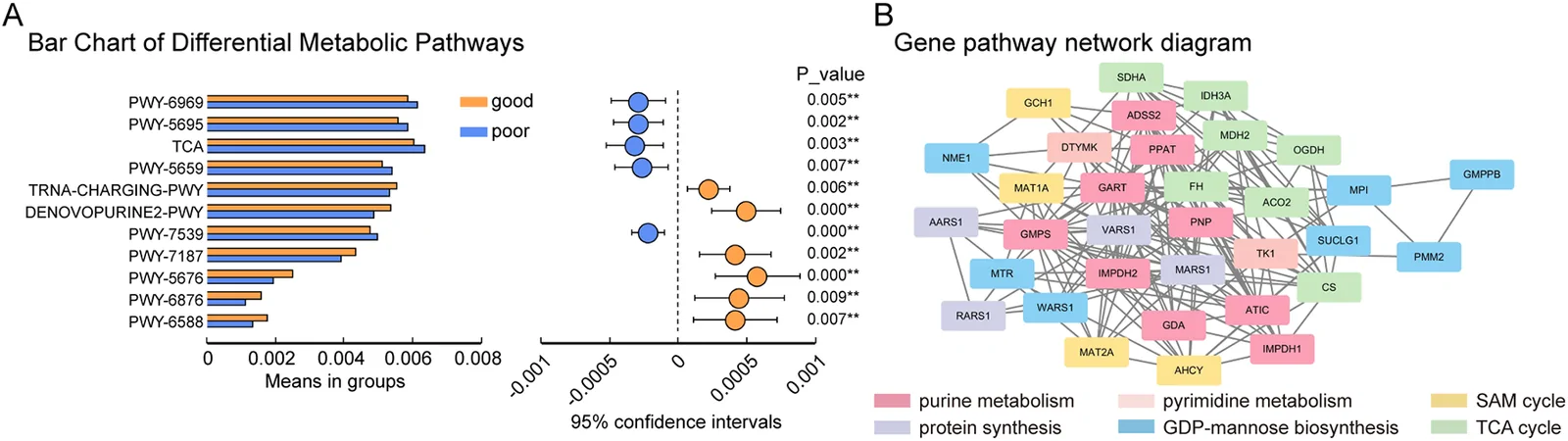
Interactions among metabolic pathways with significant differences between the good-prognosis and poor-prognosis groups and their core genes. **(A)** PICRUSt2/KEGG pathway analysis comparing the good-prognosis and poor-prognosis groups. An asterisk (*) indicates P< 0.01. **(B)** The interaction network of key genes in core metabolic pathways associated with prognosis. The network was constructed using the STRING database and visualized with Cytoscape.

Notably enriched pathways included tricarboxylic acid cycle activity (PWY-6969), *de novo* purine biosynthesis (DENOVOPURINE2-PWY), *de novo* pyrimidine biosynthesis (PWY-7187), tRNA charging, GDP-mannose biosynthesis, and S-adenosylmethionine (SAM) synthesis (PWY-6876). These functional profiles suggest enhanced biosynthetic and metabolic activity associated with aggressive tumor phenotypes.

Protein–protein interaction network analysis further identified 33 core genes linked to these metabolic pathways (**Figure 3B**). Central nodes were enriched in enzymes involved in mitochondrial energy metabolism (SDHA, OGDH, FH), nucleotide synthesis (IMPDH1, GART, ATIC, DTYMK), and amino acid metabolism and translation (GDH1, MARS1, WARS1). Collectively, these findings indicate that microbiome-associated functional pathways are linked to metabolic programs associated with glioma progression.

### 3.3 Intratumoral microbiota composition differs between low-grade and high-grade gliomas

The WHO classification, a widely adopted grading system for gliomas, exhibits a close correlation with overall survival (OS), which is also observed in our cohort (**Figures 4A–C**). Accordingly, we leveraged WHO grading levels to identify differential microbiota and develop a more precise predictive model. Microbial diversity analyses revealed significant differences between these groups.

**Figure 4:**
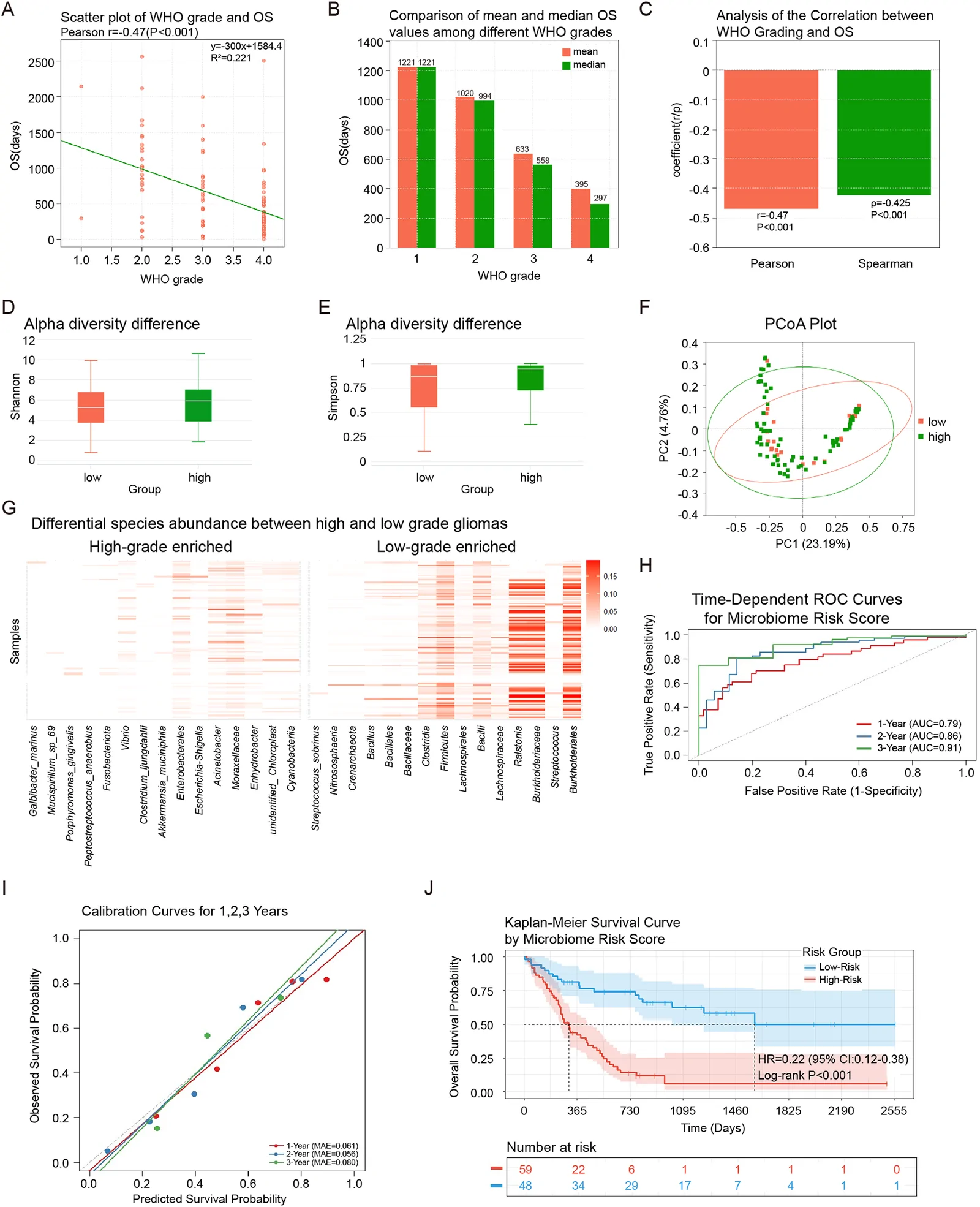
A glioma prognosis prediction model constructed based on microbe-associated factors. **(A)** Scatter plot of WHO grade and OS, Pearson r=-0.47(P<0.001). **(B)** Comparison of mean and median OS values among different WHO grades. **(C)** Pearson and Spearman correlation analysis of WHO grade and overall survival period. **(D, E)** Alpha diversity indices (Shannon and Simpson) compared between different WHO classification groups using the Wilcoxon rank-sum test. **(F)** Principal coordinate analysis (PCoA) based on Bray–Curtis distance showing beta diversity differences between groups. **(G)** Heatmap of differentially abundant taxa across different WHO classification groups. **(H)** Time-dependent ROC analysis evaluating the predictive performance of the microbiome-based stratification for overall survival. The AUC values were 0.79, 0.86, and 0.91 at 1, 2, and 3 years, respectively. **(I)** Calibration Curves for 1-, 2-, and 3-year survival. **(J)** Kaplan-Meier survival curve based on microbiome-derived risk score.

Alpha diversity analysis showed higher microbial diversity in HGG compared with LGG, as reflected by increased Shannon index values (median approximately 5.90 vs 5.25, respectively; **Figure 4D**). Similarly, Simpson index values were higher in HGG (approximately 0.94) than in LGG (approximately 0.87), indicating reduced dominance and increased community complexity in high-grade tumors (**Figure 4E**).

Principal coordinate analysis (PCoA) based on Bray–Curtis dissimilarity showed overlapping profiles between low-grade and high-grade gliomas (**Figure 4F**). PERMANOVA did not detect a statistically significant overall compositional difference (pseudo-F=1.1042, R²=0.0104, P = 0.2445; 9,999 permutations). PERMDISP also did not detect a significant difference in dispersion (F = 3.0489, P = 0.0837; **Supplementary Table 5**).

Differential abundance analysis identified distinct microbial signatures associated with tumor grade. Taxa enriched in HGG included *Galbibacter marinus*, *Porphyromonas gingivalis*, *Peptostreptococcus anaerobius, Fusobacteriota, Vibrio species, Enterobacterales, Escherichia–Shigella, Acinetobacter species, and Moraxellaceae* (**Figure 4G**). In contrast, LGG samples were characterized by enrichment of Firmicutes-related taxa, including *Clostridia, Lachnospirales, Lachnospiraceae, Bacillales, Bacillaceae, and Burkholderiales*.

These results indicate that glioma grade is associated with distinct intratumoral microbial community structures.

#### 3.3.1 Grade-associated microbiota features improve prognostic modeling

To determine whether grade-associated microbial features improve prognostic prediction, differentially abundant taxa were integrated with clinical variables. For the subset of patients with available lipid data (n = 37), random forest analysis was first applied to identify the most informative predictors. Subsequent LASSO regression revealed that *Clostridia* (coefficient = −0.1574) and *Firmicutes* (−0.0557) were retained as the strongest positively associated microbial features associated with overall survival, whereas lipid-related parameters were not selected in the final model (**Supplementary Figures 3A–D**).

These findings indicate that microbial features associated with tumor grade contribute more substantially to prognostic prediction than metabolic serum parameters in this cohort.

#### 3.3.2 Integrated WHO grade–informed model demonstrates high predictive performance

To further optimize model performance, lipid variables were excluded and analyses were extended to the full cohort (n=107). Clinical parameters, including anthropometric indices, inflammatory markers, and liver function measurements, were combined with grade-associated microbial features. Random forest screening identified the top 30 candidate predictors, which were subsequently subjected to LASSO regression.

The final model retained *Firmicutes* (-0.2806) as the strongest positively associated feature, followed by *Clostridia* (-0.1748), while age, sex, *Galbibacter marinus*, *Porphyromonas gingivalis*, *Vibrio*, BMI, and *Enterobacterales* were retained as negatively associated variables (**Supplementary Figures 3E–H**). Time-dependent receiver operating characteristic (ROC) analysis yielded apparent AUCs of 0.79, 0.86, and 0.91 at 1, 2, and 3 years, respectively (**Figure 4H**). Calibration analysis showed good agreement between predicted and observed survival probabilities (**Figures 4I, J**). After bootstrap optimism correction, the WHO-associated model yielded 1-, 2-, and 3-year AUCs of 0.7105, 0.8597, and 0.8828, respectively (**Supplementary Table 4**).

These results demonstrate that incorporation of WHO grade–associated microbial signatures substantially enhances prognostic model performance.

#### 3.3.3 Functional prediction links high-grade gliomas to nucleotide biosynthesis and metabolic remodeling

To explore functional differences associated with tumor grade, PICRUSt2-based pathway inference was performed. Comparative analysis between LGG and HGG revealed significant enrichment of metabolic pathways related to nucleotide metabolism and biosynthesis in high-grade tumors (**Figure 5A**).

**Figure 5:**
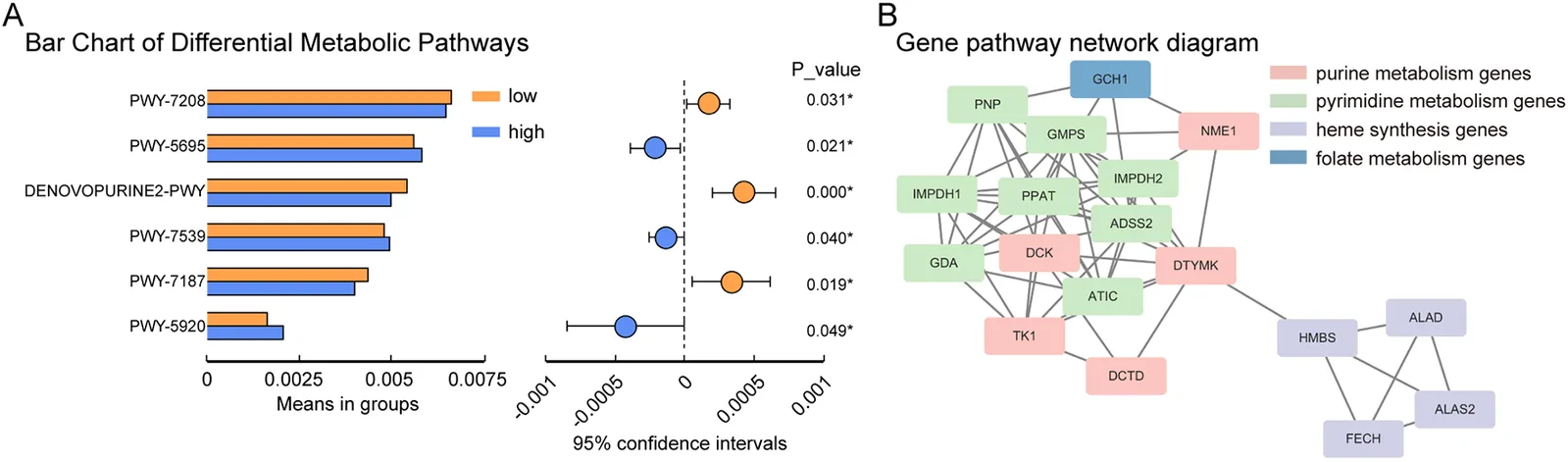
Interactions among metabolic pathways with marked differences between low-grade and high-grade gliomas and their core genes. **(A)** PICRUSt2/KEGG pathway analysis comparing low-grade and high-grade glioma groups. An asterisk (*) indicates P< 0.01. **(B)** The interaction network of key genes in core metabolic pathways associated with glioma grading. The network was constructed using the STRING database and visualized via Cytoscape.

Among these, *de novo* purine biosynthesis (DENOVOPURINE2-PWY) showed the most pronounced difference (P < 0.001), along with enrichment of pyrimidine nucleobase salvage (PWY-7208), *de novo* pyrimidine biosynthesis (PWY-7187), inosine 5’-phosphate degradation (PWY-5695), and glycine heme biosynthesis (PWY-5920).

Network analysis of core genes associated with these pathways demonstrated a highly interconnected metabolic architecture centered on purine and pyrimidine metabolism (**Figure 5B**). Genes involved in nucleotide biosynthesis occupied central network positions, highlighting nucleotide metabolic pathways as prominent functional features associated with high-grade glioma biology.

## 4 Discussion

### 4.1 Intratumoral microbiota signatures are associated with glioma prognosis and metabolic remodeling

In this study, we found that intratumoral microbiome composition is closely associated with glioma prognosis and tumor grade. By integrating microbial and clinical variables, we identified *Firmicutes* and *Clostridia* as positively associated features, whereas taxa including *Porphyromonas gingivalis, Alphaproteobacteria, Galbibacter marinus, and Enterobacterales* were associated with unfavorable clinical outcomes. These findings suggest potential prognostic associations but do not establish protective or pathogenic functions, or incremental predictive value beyond established clinicopathological factors.

Previous studies have shown that *Porphyromonas gingivalis* promotes tumor cell proliferation through gingipain-mediated activation of MAPK signaling and sustained inflammatory responses (Gnanasekaran et al., 2020; Mu et al., 2020). Chronic microbe-driven inflammation is increasingly recognized as a contributor to tumor progression and immune evasion (Zhao et al., 2021). Recent studies focusing on the gut–brain microbiome axis and microbiota-associated mechanisms in glioblastoma further highlight the importance of microbial-host interactions in shaping tumor biology, treatment response, and clinical outcomes (Ganesh et al., 2025). In contrast, certain microbial taxa have been reported to exert tumor-suppressive effects through metabolic or immunomodulatory mechanisms (Adorisio et al., 2019; Narunsky-Haziza et al., 2022). Although *Firmicutes* and *Clostridia* have not been extensively studied in glioma, their consistent association with favorable prognosis in our cohort suggests a potential protective role that warrants further functional investigation.

Functional prediction analysis revealed enrichment of metabolic pathways related to energy metabolism and nucleotide biosynthesis in poor-prognosis tumors. In particular, enrichment of *de novo* purine biosynthesis pathways suggests increased biosynthetic demand associated with rapid tumor proliferation. Key enzymes within these pathways, including IMPDH1 (Zhang et al., 2023) and GART (Tang et al., 2023), have previously been linked to cancer cell growth and therapeutic vulnerability. Together, these observations suggest potential associations between intratumoral microbial signatures and metabolic pathway alterations observed in aggressive gliomas.

Importantly, while our data establish strong associations between microbial signatures and clinical outcomes, they do not demonstrate causality. Experimental validation using controlled *in vivo* and *in vitro* systems will be required to determine whether specific microbial taxa directly influence tumor progression or primarily reflect tumor microenvironmental changes.

### 4.2 WHO grade–associated microbial patterns highlight links between malignancy and biosynthetic metabolism

We further found that gliomas of different WHO grades harbor distinct intratumoral microbial profiles. High-grade tumors exhibited enrichment of taxa commonly associated with pro-inflammatory or pathogenic phenotypes, including *Enterobacterales, Vibrio species, and Porphyromonas gingivalis*, whereas low-grade tumors were characterized by Firmicutes- and Clostridia-dominated communities. Integration of these grade-associated microbial features substantially improved prognostic model performance.

Functional inference revealed marked enrichment of nucleotide metabolism pathways in high-grade tumors, including *de novo* purine and pyrimidine biosynthesis. These metabolic programs are involved in processes related to DNA replication and cell division, suggesting that metabolic pathway alterations may be associated with glioma malignancy. Network analysis further highlighted nucleotide biosynthesis enzymes as highly connected nodes, suggesting a potential association between biosynthetic capacity and high-grade disease.

Although prior studies have reported that Enterobacterales-derived genotoxins and inflammatory mediators can induce DNA damage in other cancer types (Nougayrède et al., 2006; Liu et al., 2022), direct mechanistic evidence in glioma remains limited. Our findings support that intratumoral microbial communities may be linked to genomic instability and metabolic stress responses, but additional experimental studies will be required to clarify these relationships.

### 4.3 Clinical implications: microbiome-informed stratification and therapeutic opportunities

Current glioma diagnosis and grading rely primarily on histopathology, molecular markers, and neuroimaging. However, these approaches incompletely capture tumor heterogeneity and treatment responsiveness. Our data suggest that intratumoral microbiota profiling may provide complementary prognostic information that could enhance risk stratification and clinical decision-making.

From a therapeutic perspective, temozolomide remains the backbone of glioma chemotherapy, yet resistance frequently limits its long-term efficacy (Lee, 2016; Hou et al., 2023; Jiapaer et al., 2018). Increasing evidence indicates that microbiota-derived metabolites influence responses to chemotherapy and radiotherapy (Cheng et al., 2020; Alpert et al., 2021). For example, short-chain fatty acids such as butyrate have been shown to suppress glioma cell proliferation and enhance radiosensitivity in experimental models (Entin-Meer et al., 2005). Similarly, modulation of microbial composition has been reported to improve treatment efficacy in other cancer types (Hou et al., 2021; Feng et al., 2023; Lu et al., 2024).

Our findings raise the possibility that microbiome-informed strategies, including dietary interventions, probiotic supplementation, or microbiota-targeted therapies, may complement existing glioma treatment paradigms. However, such approaches must be applied cautiously, as indiscriminate microbial manipulation may disrupt beneficial microbial communities and immune homeostasis.

Importantly, inter-patient heterogeneity is likely to influence therapeutic responsiveness. Our results suggest that microbiota-based interventions may be most effective in patients exhibiting adverse intratumoral microbial signatures.

Collectively, these findings suggest potential associations between intratumoral microbial signatures and glioma prognosis that warrant further investigation. Integration of microbiome profiles with molecular classification, immune characteristics, and metabolic features may further improve our understanding of glioma heterogeneity and provide a foundation for future microbiome-informed therapeutic strategies.

### 4.4 Study limitations and future directions

Several limitations should be acknowledged. First, this study has limitations in the integration of key molecular markers. While we incorporated critical molecular features including IDH and MGMT, only 24 samples had complete data, leading to insufficient statistical power and a high risk of bias in the constructed predictive model. Nevertheless, these molecular markers are of paramount importance for glioma prognosis. Therefore, future prospective studies are warranted to expand the cohort and systematically integrate multi-omics data, with the aim of developing more precise prognostic models. Second, detailed information regarding extent of surgical resection and performance status was unavailable for a subset of patients, limiting adjustment for these important prognostic variables. Third, We cannot exclude the possibility that part of the detected microbial signals may be influenced by reagent- or environment-derived contamination, particularly in low-biomass FFPE samples. Lastly, functional predictions based on PICRUSt2 are indirect and computationally inferred and therefore require validation by metabolomic or experimental approaches.

The detection of microbial DNA in FFPE glioma specimens does not establish the presence of viable bacteria or their spatial localization within tumor tissue. Future prospective studies incorporating standardized clinical data collection, spatial microbial validation, and multi-omics integration will be necessary to confirm the generalizability of our findings. In addition, mechanistic studies using orthotopic glioma models and controlled microbial perturbation approaches will be essential to establish causal relationships between specific microbial taxa and tumor biology.

Despite these limitations, our study suggests that intratumoral microbiota signatures are associated with glioma malignancy, metabolic pathway alterations, and patient prognosis, highlighting their potential value as biomarkers and warranting further investigation of their biological and clinical relevance.

## 5 Conclusion

In this study, we found that intratumoral microbiota composition is associated with glioma malignancy and patient prognosis. By integrating microbial signatures with clinical and functional pathway analyses, we identified microbiome-derived features that may provide complementary prognostic information in addition to conventional clinicopathological parameters.

These findings expand current understanding of the glioma tumor microenvironment and highlight the potential value of incorporating microbiome profiling into multi-omics–based diagnostic and risk stratification frameworks. Moreover, the observed associations between microbial signatures and metabolic pathway alterations suggest potential directions for future microbiome-informed therapeutic investigation.

Future studies focusing on mechanistic validation and prospective clinical evaluation will be essential to translate these observations into practical applications. Collectively, our results are consistent with the emerging concept that host–microbiota interactions represent an important biological dimension of glioma progression and may have relevance for the development of more precise and personalized treatment approaches.

## Data Availability

The sequencing data presented in this study have been deposited in the NCBI Sequence Read Archive (SRA) under BioProject accession PRJNA1543727.
