# Supplementary material for: Intratumoral microbiota stratifies glioma malignancy and patient survival through metabolic associations
Source: Front Cell Infect Microbiol. 2026 Oct 23;16:1940097. doi: 10.3389/fcimb.2026.1940097 (PMC13739529; doi:10.3389/fcimb.2026.1940097)
Supplement: Supplementary file 1 [file SupplementaryFile1.docx]

## **Supplemental Material**

**Supplemental Fig. 1. Distribution of the Top 10 Species by Abundance in 107 Glioma Samples.**

(A) Phylum level. (B) Class level. (C) Order level. (D) Family level. (E) Genus level. (F) Species level

**Supplemental Fig. 2. Lasso Regression Analysis Based on Lipid Profile and OS Grouping.**

(A) The top 15 factors identified by random forest analysis regarding the association between blood lipid-related clinical factors and OS. (B) Lasso regression coefficient path plot. (C) Lasso regression cross-validation curve for selecting the optimal penalty parameter λ. When lambda.min is selected, λ = 2. (D) Multivariate Cox regression analysis of the non-zero coefficient variables selected by Lasso. Positive coefficients indicate protective factors, and negative coefficients indicate risk factors.(E) The top 30 factors related to OS identified by random forest analysis. (F) The path diagram of Lasso regression analysis associated with OS.(G) The cross - validation plot of Lasso regression analysis related to OS. When lambda.min is selected, λ = 7. (H) The non - zero coefficient variables selected by Lasso regre ssion associated with OS. Positive values represent protective factors, while negative values represent risk factors. Specifically, Firmicutes = - 0.2463, Age = 0.2108, Clostridia = - 0.1300, Gender = 0.1136, Galbibacter_marinus = 0.0367, Alphaproteobacteria = 0.0246, Porphyromonas_gingivalis = 0.0031.

**Supplemental Fig. 3. Lasso Regression Analysis Based on Lipid Profile and WHO Grading Grouping.**

(B) The top 15 factors identified by random forest analysis regarding the association between blood lipid-related clinical factors and OS. (B) Lasso regression coefficient path plot. (C) Lasso regression cross-validation curve for selecting the optimal penalty parameter λ. When lambda.min is selected, λ = 2. (D) Multivariate Cox regression analysis of the non-zero coefficient variables selected by Lasso. Positive coefficients indicate protective factors, and negative coefficients indicate risk factors.(E) The top 30 factors identified by random forest analysis in relation to WHO grading. (F) The path diagram of Lasso regression analysis associated with grading.(G) The cross - validation plot of Lasso regression analysis related to grading. When lambda.min is selected, λ = 9. (H) The non - zero coefficients of Lasso regression associated with WHO grading: Firmicutes = - 0.2806, Age = 0.2424, Clostridia = - 0.1748, Gender = 0.1475, Galbibacter_marinus = 0.1133, Porphyromonas_gingivalis = 0.0561, Vibrio = 0.0277, BMI = - 0.0243, Enterobacterales = 0.0009.
